# Cities of the Anthropocene: urban sustainability in an eco-evolutionary perspective

**DOI:** 10.1098/rstb.2022.0264

**Published:** 2024-01-01

**Authors:** Marina Alberti

**Affiliations:** Department of Urban Design and Planning, University of Washington, Seattle, WA, 98195, USA

**Keywords:** eco-evolutionary dynamics, urban evolution, complex systems, evolutionary potential, urban sustainability

## Abstract

Cities across the globe are driving systemic change in social and ecological systems by accelerating the rates of interactions and intensifying the links between human activities and Earth's ecosystems, thereby expanding the scale and influence of human activities on fundamental processes that sustain life. Increasing evidence shows that cities not only alter biodiversity, they change the genetic makeup of many populations, including animals, plants, fungi and microorganisms. Urban-driven rapid evolution in species traits might have significant effects on socially relevant ecosystem functions such as nutrient cycling, pollination, water and air purification and food production. Despite increasing evidence that cities are causing rapid evolutionary change, current urban sustainability strategies often overlook these dynamics. The dominant perspectives that guide these strategies are essentially static, focusing on preserving biodiversity in its present state or restoring it to pre-urban conditions. This paper provides a systemic overview of the socio-eco-evolutionary transition associated with global urbanization. Using examples of observed changes in species traits that play a significant role in maintaining ecosystem function and resilience, I propose that these evolutionary changes significantly impact urban sustainability. Incorporating an eco-evolutionary perspective into urban sustainability science and planning is crucial for effectively reimagining the cities of the Anthropocene.

This article is part of the theme issue ‘Evolution and sustainability: gathering the strands for an Anthropocene synthesis’.

## 1. Introduction

Global urbanization is a prominent feature of the Anthropocene, driving a critical transition in the dynamics of human–Earth systems and challenging us to rethink the city in the context of planetary change. While humans have been altering ecosystem processes for millennia, the emergence and rapid development of cities across the globe represents a major shift in human–nature relationships, leading to the relatively recent discontinuity in both the intensity and scale of planetary human-driven ecological transformation [1,2].

Urbanization is driving systemic change in social and ecological systems and altering Earth's ecosystems by causing a new wave of space–time compression [3], accelerating the rates of interactions among people and places, and multiplying the numbers and strengths of connections between human activities and Earth ecosystems [4]. The effects of the multiple changes set in place by the urban transition are reflected in the rapid increase in resource extraction, greenhouse gas emissions and land conversion, disrupting the climate system and rapidly reducing biodiversity, thereby changing the interactions between ecological and evolutionary processes that maintain life [5,6].

Increasing evidence shows that cities are changing the genetic and cultural makeup of many populations, including animals, plants, fungi and microorganisms, which might have significant effects on socially relevant ecosystem functions such as nutrient cycling, pollination, water and air purification and food production both locally and globally [7,8]. Urban-driven evolutionary changes in species traits affect ecosystem dynamics by altering population dynamics, shaping species interactions and altering community assembly in diverse metacommunities [9]. Eco-evolutionary feedback—the reciprocal interaction between ecology and evolution—can affect ecosystem stability and resilience, and hence influence sustainability over the long term [10,11]. Rapid evolution determines how organisms and ecosystems respond to human-caused pressures such as habitat loss, the introduction of new species, climate change, as well as to conservation efforts.

The study of biodiversity in urban landscapes has made significant progress, shedding light on the multifaceted interplay between urbanization and ecological [12–15] and evolutionary change [16–18]. Remarkable progress has been made in understanding the complex interactions governing these systems [19,–21] and their variability across scale [22]. These new insights are shaping nuanced conservation strategies that embrace the complexity inherent in urban biodiversity [23]. In a notable step, the 15th Conference of Parties to the United Nations Convention on Biological Diversity, a new platform identifies the maintenance of genetic diversity within populations as a key factor for safeguarding their adaptive potential [24]. However, despite increasing evidence of urban evolutionary change and calls for deliberate management of anthropogenic evolution to address global challenges [25–30], current approaches to urban sustainability often overlook these dynamics.

In this review, I argue that integrating evolutionary principles into the planning and design of cities is critical for achieving urban sustainability. First, I provide an overview of the socio-eco-evolutionary transition that has arisen as a result of global urbanization. I propose that urbanization catalyses a systemic transformation in the interactions among social and ecological systems shaped by technological and institutional factors [4]. This shift gives rise to the *emergence* of distinctive ecological properties of urbanizing regions, which *speed up* evolutionary change in species that contribute to vital ecosystem functions and tighten eco-evolutionary *coupling*, altering the *resilience* that enables local and global sustainability over the long term.

**Figure 1.**
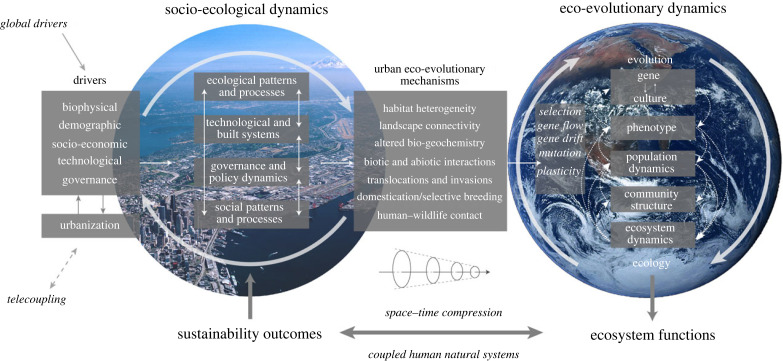
A conceptual framework linking socio-ecological and eco-evolutionary dynamics of coupled human–natural systems. Key natural and anthropogenic drivers of change (e.g. climate, demographics, economics and policy) influence eco-evolutionary dynamics and its feedback through interactions among ecological, technological, governmental and social system components of the urban ecosystem. Highlighted are the emerging mechanisms of how global urbanization drives eco-evolutionary dynamics and feedback affecting ecosystem function and sustainability outcomes by altering habitat heterogeneity, landscape connectivity, biogeochemistry, biotic and abiotic interactions, translocations and invasions, domestication and selective breeding and human–wildlife contact.

Drawing on Holling's [31] concept of resilience and Gunderson's [32] and Sgrò *et al*.'s [25] definitions of evolutionary resilience and building upon the distinction made by Elmqvist *et al*. [33] between resilience and sustainability, I define urban resilience as the inherent property of a social-ecological system that enables it to maintain its essential functions in the face of dynamic changes. Sustainability is a normative concept that requires meeting the present's needs while preserving Earth's life-support systems. Using evidence of observed changes in traits that play a significant role in maintaining ecosystem functioning and resilience, I identify emerging mechanisms linking urbanization to eco-evolutionary dynamics and the potential feedback to sustainability outcomes (figure 1) [9,34,35].

Building on this foundation, I explore how eco-evolutionary dynamics within cities are shaped by interactions among various selective agents within unique urban spatial arrangements, the result of interplay among human activities, infrastructure development and regional ecological variations. Distinctive eco-evolutionary signatures observable across variable patterns of urbanization and across scales suggest that the design of cities plays a critical role in preserving the evolutionary processes that underlie urban resilience. Focusing on green and blue urban infrastructure (GBI), I provide evidence of the interactions between GBI and the evolution of species traits that maintain ecosystem function and resilience. Incorporating evolutionary perspectives into urban planning can enhance species' adaptive potential, thereby strengthening urban ecosystems and promoting their sustainability. I propose principles for embedding evolution into the design of sustainable urban development strategies.

## 2. The urban socio-eco-evolutionary transition

Cities are unquestionably the most emblematic signature of the Anthropocene. It is widely known that with more than 4.5 billion people, more than 56% of the world population, urban areas today generate more than 80% of the world's economy and account for over 70% of global energy use and energy-related greenhouse gas emissions [36,37]. Global urbanization is rapidly expanding in terms of population size and land areas [38,39]. What is less known is how the variable spatio-temporal relationship between population and built-up land relates to the social and physical drivers of urbanization across diverse regions and scales. This dynamic plays a critical role in shaping the interactions between urbanization and environmental change [40].

Over the past century, a significant shift has occurred in the dominant configuration of urban regions, evolving from centralized settlements to polycentric structures, ultimately leading to networked megaregions [36,41]. This transformation is the outcome of changes in function from industrial to service- and knowledge-based economies, combined with advancements in infrastructure and technology, fostering enhanced regional and global connectivity and interdependence [42].

The emergent urban megaregions represent networks of metropoles, characterized by a multi-nodal mosaic of developed and undeveloped land that breaks down the traditional boundaries between cities, regions and suburbs giving rise to a new local and global landscape [43]. Urban megaregions, such as the Boston–Washington corridor in the United States (US), the Paris–Amsterdam–Brussels–Munich region in Europe, the Pearl River Delta in China and the Tokyo–Yokohama region in Japan, are densely populated and contribute significantly to their national economies. For instance, North American megaregions, primarily located in the US, house 70% of the US population, generate 75% of the US economy and emit carbon at rates comparable to those of entire nations [36,41].

These changes in regional urban structures have a major impact on the effects of urbanization on socio-ecological dynamics and eco-evolutionary change, influencing both local and global sustainability through processes such as land conversion, habitat alteration, changes in biotic interactions, increased frequency and intensity of disturbances and the emergence of new ones [42]. Urban megaregions, characterized by high population densities, resource concentration and central roles in trade and logistics, can either exacerbate or mitigate the environmental footprint of global production and consumption systems. These regions accelerate eco-evolutionary changes by increasing resource demand, modifying land management and facilitating the translocation of non-native species, extending their impacts beyond their physical boundaries.

At the same time, the high population density and human activity associated with these urban agglomerations bring the environmental impacts of this growth into sharp focus, often prompting efforts to address these issues. In examining innovative governance in Tianjin, London and Bangkok, Doran *et al*. [44] show that access to the resource diversity of megaregions—including economic, knowledge and social capital—can encourage beneficial cross-sector collaborations to reduce megaregions' reliance on imported resources. However, owing to their polycentric development, inherent governance challenges could trigger large-scale collective action problems with the potential to undermine these benefits.

I propose that the rapid development of urban regions, their distinctive spatial structure (resulting from the interactions of human agency, constructed infrastructure and the physical environment), and the simultaneous occurrence of numerous disturbances set the eco-evolutionary dynamics of urban ecosystems apart from natural and other anthropogenic systems.

### (a) Emergence

**Figure 2.**
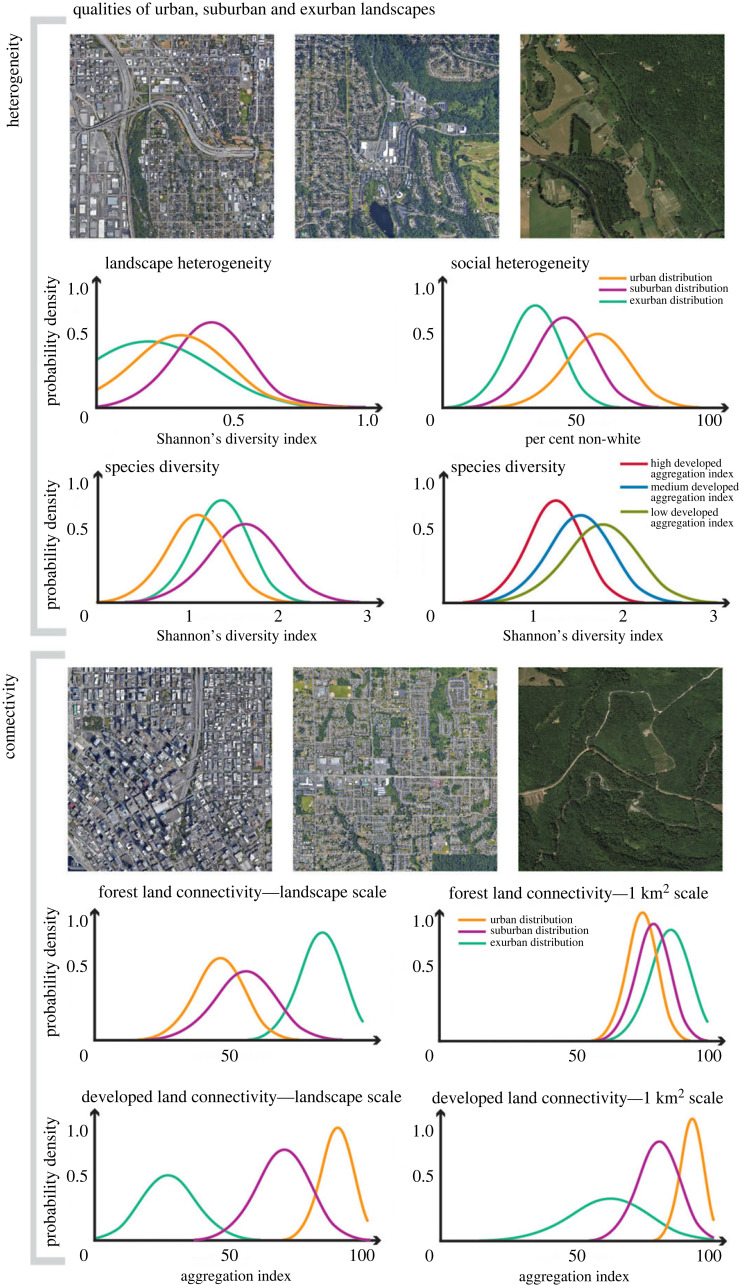
Hypothesized patterns across urban, suburban and exurban areas. Urban areas are hypothesized to have intermediate landscape heterogeneity, lowest species diversity, highest social heterogeneity and developed land connectivity, with least forest connectivity. A potential inverse correlation is hypothesized between species diversity and the Aggregation Index of development [45]. These patterns, hypothesized to be more clustered at the square-kilometre scale, differ in suburban areas with peak landscape heterogeneity, species diversity and moderate connectivity levels, and in exurban areas with minimal landscape heterogeneity and developed land connectivity, but high forest connectivity. Inset aerial images in the first panel illustrate the varying degrees of landscape heterogeneity across a hypothetical gradient of urbanization, while those in the second panel show examples of forest and developed land connectivity.

Urban landscapes are structurally complex and exhibit unique heterogeneity and connectivity, emerging from complex social-technological and ecological interactions, which create unique species community assembly and selection gradients (e.g. temperature, fragmentation and pollution) that alter metacommunities and eco-evolutionary dynamics [21] (figure 2). Landscape heterogeneity in cities is unique because of a combination of natural and engineered elements and the socio-cultural characteristics and behaviours of individuals and institutions [46]. Although urban heterogeneity can potentially promote diverse species with unique niches, the composition of the urban species pool is ultimately dictated by environmental filters associated with urban land use [47,48], which may lead to biotic homogenization [49,50].

Variable social and ecological heterogeneity in urban landscapes is the result of diverse historical development patterns, a combination of natural and engineered landscape elements, and the socio-economic characteristics and behaviours of individual people, communities and institutions. Development decisions, management choices and individual preferences can alter landforms, biophysical and ecological networks and the heterogeneity of nutrients, materials and water cycling. The interplay between neighbourhood socio-economic stratification, governance structure and planning practises often shapes ecological variation across urban landscapes and creates unequal distributions of ecological resources and access to ecosystem services among socio-economically diverse communities and neighbourhoods [51].

Urbanization also fundamentally rewires connectivity, differently affecting social and ecological communities and their interactions. By reshaping social and ecological networks, cities alter the social, ecological and evolutionary processes that maintain their resilience and adaptive capacity. Urbanization isolates previously connected habitat patches, subpopulations and species, while simultaneously connecting those that were previously isolated from each other [18,52,53]. By intensifying social interactions within and across cities, urbanization expands eco-evolutionary changes [7,8] and creates new inequalities [4], while simultaneously stimulating social innovation [54]. Understanding how patterns of urban development affect eco-evolutionary dynamics will provide critical insights for designing and planning urban systems and infrastructure schemes that align with ecological and evolutionary processes that support sustainability.

### (b) Speed

Studies show that evolution is faster in the city than in surrounding areas owing to both strong selection pressures and novel ones [7,8]. Through a meta-analysis of experimental and observational studies reporting more than 1600 phenotypic changes in species across multiple regions, my colleagues and I [7] discriminated an urban signature of phenotypic change beyond established natural baselines and other anthropogenic signals. We show a clear urban signal: rates of phenotypic change are greater in urbanizing systems than in natural and non-urban anthropogenic systems. The interactions of multiple selection pressures (habitat modification, introductions, fragmentation, pollution, novel disturbances, etc.) can also lead to stronger eco-evolutionary feedback. The urban signal of phenotypic change observed across the globe (e.g. greater phenotypic change in urbanizing systems compared with natural and non-urban anthropogenic systems) may be the result of multiple influences that the urban environment imposes on organisms, which can increase the total strength of selection on a trait, the number of traits under selection or both.

Several factors might account for the speed at which evolution occurs in urban environments. Organisms’ size and metabolic rates are potential determinants of evolutionary speed. The role of urban heat islands in accelerating the evolution of ectotherms in cities remains an intriguing area of investigation [55]. A rapid pace of change can create a moving evolutionary target, which can accelerate the pace of adaptation. The density of interactions of both human and non-human organisms between different species or between organisms and their environments, can create new niches and therefore new opportunities for evolutionary innovation. Cities' high environmental variability, both spatially (e.g. differences between a park and a parking lot) and temporally (e.g. changes in noise or light pollution throughout the day), can lead to stronger selective pressures, potentially catalysing faster evolution. Some studies suggest that urban areas might be hotspots of evolutionary change, where new traits and new species arise at a faster rate than in other environments [7,8]. This could be owing to a combination of the factors mentioned above as well as the unique challenges and opportunities presented by urban life.

### (c) Coupling

Urbanization amplifies the feedback between ecology and evolution by simultaneously altering both processes and their interconnections, with ecological and evolutionary variables reinforcing each other [56]. Cities influence the evolution of numerous species, altering their interactions and changing demographic rates (such as reproduction, survival or dispersal). This can either amplify eco-evolutionary feedback through directional selection on common traits or dampen selection strength by easing survival and reproduction conditions, or making certain traits less advantageous [57].

The interplay between altered gene flow and variation in selection pressures can alter trait matching in ecological networks, including predator–prey (e.g. beak size matching seed shape), parasite–host, competitive and mutualistic interactions (e.g. flower shape matching bee's proboscis), with potential consequences for ecosystem function [58,59]. Urbanization reduces top-down control in food webs by increasing the effect of bottom-up mechanisms (i.e. energy and nutrient supply) through the greater availability of anthropogenic resources (e.g. food resources) [60,61]. The shift from top-down to bottom-up processes may feed back on population and community demographics, relaxing and/or reinforcing selection pressures, and potentially strengthening eco-evolutionary feedback through increasing directional selection at various trophic levels. Investigating urban-driven shifts in network structure can illuminate the effects of urbanization on evolutionary potential, ecosystem function and resilience [62].

### (d) Resilience

Resilience in urban ecosystems is governed by complex interactions among multiple social and ecological processes that maintain long-term ecosystem function. Cities are coupled socio-technological and ecological systems, the product of co-evolving human and natural systems mediated through technology [63]. Achieving sustainability requires understanding the interplay between a city's socio-technological and ecological dynamics that affect ecosystem function and resilience at the local and global scales [64]. Furthermore, the cross-boundary independence of urban infrastructure—such as food systems, energy, transportation and sanitation—through regional and global networks underscores the far-reaching effects of actions within a socio-ecological system [65].

Urbanization affects ecological and evolutionary processes across scales, with city size and structure contributing significantly to this variability [9,22]. At smaller scales, it tends to simplify ecosystems [66], while interactions between human and ecological systems at larger scales add complexity, creating novel conditions shaped by cultural, socio-economic and political contexts [67]. Understanding this variability is crucial for designing sustainable strategies, as sustainability drivers—social, ecological and technological—depend on scale. Scaling theories aim to unravel the universal principles governing city function [68–70], revealing diverse patterns—linear, superlinear and sublinear—in various social and physical attributes, as they relate to population size [69]. However, underlying mechanisms linking city scale to biodiversity may account for marked variations in socio-ecological outcomes across cities that differ in size. Uchida *et al*.'s [22] investigation into the scaling relationships of urban biodiversity illuminates how the interplay among environmental factors, human influences and socio-economic and eco-evolutionary drivers can produce both linear and nonlinear relationships across scales.

Urban resilience is profoundly shaped by historical contingencies and long-term socio-ecological interactions. Today's rapidly evolving urban environments bear the enduring imprints of past land-use decisions. For instance, the historical displacements of Indigenous people in the US owing to urban expansion and the legacy of last century's segregationist land-use policies continue to influence current socio-ecological and eco-evolutionary dynamics [71]. Moreover, past infrastructure transformations, such as early twentieth century hydroelectric dams, have left indelible imprints on the urban landscape and its ecological processes [72].

An important dimension of resilience is the ability of ecosystems to reorganize and renew [31,73]. The convergence of significant societal, technological and environmental transitions with global urbanization offers a unique opportunity for systemic change, potentially catalysing the transition to a sustainable socio-ecological system by accounting for its complexity and ability to evolve. Recent initiatives that integrate sustainable systems and GBI with social equity considerations represent a new understanding of the interdependence between social and ecological resilience, which could lead to more effective sustainability transitions. To fully realize this potential, an eco-evolutionary perspective must be incorporated into the design of equitable and effective sustainability strategies. Evolutionary resilience acknowledges the crucial role of genetic diversity and evolutionary processes in shaping community ecology and the capacity of ecosystems to adapt to changing conditions [32].

## 3. Mechanisms linking urban eco-evolutionary dynamics to sustainability

Urbanization plays a critical role in shaping eco-evolutionary dynamics and feedback mechanisms that affect ecosystem function and sustainability outcomes (figure 1). Over the past decade, extensive research on urban-driven contemporary evolution has documented a range of phenotypic changes—some confirmed to be evolutionary—in species traits across diverse taxa worldwide, contributing to ecosystem resilience and long-term sustainability [7,8,34]. Cities have emerged as a major focus of study in evolutionary biology, as scholars recognize the opportunities cities offer to explore evolution in real time in globally replicated experiments to address unanswered questions about the repeatability and predictability of evolution [74]. Despite their heterogeneity, cities provide a unique opportunity to ask whether populations and taxa exposed to similar selection pressures tend to undergo similar evolutionary changes and how the co-occurrence of multiple selection pressures may alter (limit or strengthen) adaptation to individual stressors or lead to synergistic interactions that may amplify or dampen eco-evolutionary feedback [11]. At the same time, the great worldwide variability of city structure and city size offers a unique opportunity to disentangle the properties of cities that affect evolutionary outcomes.

Cities provide a novel context for evolutionary studies that presents both new opportunities and new challenges [62]. The dominant presence of humans and their societies introduces multiple layers of complexity into ecological and evolutionary processes [9]. A crucial question is whether the framework and assumptions that govern eco-evolutionary dynamics in natural systems can be applied to coupled urban human–natural systems. Integrating social, technological and governance drivers of evolution and eco-evolutionary feedback presents new insights into the study of evolution but demands a thorough understanding of the complex and unique urban context in which evolution occurs [9,34,35]. Rather than reiterating existing research findings (numerous reviews of human impacts on evolution in urban contexts have been published [7,8,34,35,75]), I focus on how systemic properties (multiple agents, variability, interactions, space–time compression and scale) resulting from the coupling of human and natural systems and the interactions among social, institutional, technological and ecological factors modify the drivers of eco-evolutionary change that affect urban sustainability.

### (a) Multiple agents of selection

Urbanization alters the dynamics of the Earth's ecosystems by reshaping the ecological and evolutionary processes that sustain genetic diversity. The effects of the urban transition on eco-evolutionary dynamics result from mutation, genetic drift, gene flow and natural selection, all of which can alter allele frequencies within and across populations. However, the complex nature of urban environments makes it challenging to disentangle the specific mechanisms driving urban evolution, leading to studies that report inconsistent outcomes [16,17].

While most evolutionary studies have focused primarily on urban adaptive evolution, neutral evolutionary processes can also influence eco-evolutionary feedback. Mutations are a fundamental source of genetic variation and have been found to occur in response to urban pollution [76–78], though it remains uncertain whether the urban environment itself elevates mutation rates. Urban adaptation typically arises from pre-existing allelic diversity or standing genetic variation within populations [79]. Urbanization leads to population declines that exacerbate the effects of genetic drift, reducing genetic diversity within populations and increasing differentiation among populations [80]. However, urbanization can also boost regional genetic diversity by creating novel habitats and establishing new ecological networks that facilitate population expansion and enhance connectivity, thereby decreasing genetic drift [81]. The effect of urbanization on dispersal and gene flow remains unclear, with studies showing contrasting findings [81]. Urban landscapes can introduce artificial barriers that isolate populations but also create new corridors that may bring previously isolated populations and species together, leading to varying effects on dispersal and gene flow [16].

Emerging hypotheses of the mechanisms linking urbanization to eco-evolutionary change are based on evidence that patterns of urban development and infrastructure affect natural habitats, biogeochemistry and biotic interactions along multiple axes in subtle ways [9]. Cities act as agents of selection through several mechanisms. Land use, buildings and roads fragment habitats, reducing gene flow and diversity [16]. Air pollution selects for organisms that can handle high stress [82]. Water and soil contaminants favour tolerant species [83,84]. Urban heat islands can lead to the evolution of higher heat tolerance [55,84]. Artificial light and noise can alter circadian rhythms and life-history traits [85]. Transportation can disperse genes among populations. Urban green infrastructure provides habitats and corridors for gene flow [16]. Landscaping and non-native species affect biotic interactions and cause evolutionary changes in native species [86]. Altered water and season length affect the life-history traits of organisms [87]. Urban eco-evolutionary studies have focused on single disturbances; however, it is unclear whether organisms adapt to specific pressures (e.g. heat) or multiple pressures (e.g. heat and pollution). It is also unknown whether multiple selection pressures and their spatial interactions hinder or enhance adaptation to individual stressors [34].

### (b) Pattern variability across scales

Urban environments exhibit variable heterogeneity and connectivity influenced by historical contingencies that affect evolutionary processes across scales. Urban landscapes are mosaics of multiple stressors that act on diverse organisms through different processes, leading to nonlinear responses in populations and communities. These mechanisms and their effects are not uniform or scale invariant across landscapes and urban regions. The multidimensional nature of urban disturbances and co-occurrence of multiple stressors can cause synergistic effects, leading to a large number of possible scenarios [20,88].

Development patterns create distinct landscape signatures and temporal shifts in ecosystem processes that affect species composition, community assembly and evolutionary potential. The spatial configuration of urban development in cities affects eco-evolutionary interactions by changing social and ecological heterogeneity and connectivity; the impact depending on scale [9]. At smaller scales (microscale), urbanization may reduce ecological heterogeneity [15], simplifying ecosystem complexity by eliminating processes, landforms, species and sources of fine-scale ecology that maintain resilience [66]. At the landscape mesoscale, urbanization generates varied biophysical conditions, processes and temporal dynamics influenced by human preferences, opportunities and behaviours. Land use decisions, management choices and individual preferences can shape landforms, physical networks, nutrient distribution, material cycling, water cycling and species interactions [89]. The variability of environmental pollution (e.g. night lights, atmospheric emissions and noise) is also time-dependent owing to the variability and timing of human activities.

At the macro scale, such as the metropolitan or regional scale, consistent patterns of urban development and habitat fragmentation can be observed, influenced by cultural and historical legacies as well as climate [90]. Structural inequalities across the urban landscape drive ecological outcomes, often generating socio-economically distinct gradients of land cover and exposure to pollutants [71]. Social and ecological heterogeneity in urban environments interact across time and space as drivers and outcomes of biophysical and social processes, affecting future systems state and functions [91]. Changes in spatial and temporal heterogeneity, along with a reduction in habitat quality, may generate asymmetrical selective pressures favouring certain species and traits, potentially leading to ecological homogenization [50].

### (c) Socio-ecological interactions

The urban transition fundamentally reshapes the interplay between social and ecological systems setting the stage for novel socio-eco-evolutionary dynamics [9,35]. Complex interactions resulting from changes in habitat and species composition, coupled with emerging spatial and temporal patterns of resource availability, might produce new trophic dynamics (i.e. shifts in control from top-down to bottom-up) [60], altering the ecological networks of interacting species and their evolutionary potential [9]. Urban ecological networks may become less diverse and less complex. However, because specialists are lost more quickly than generalists, species richness may decline faster than the number of interactions [58,92]. Thus, the structure of urban networks is more nested (specialists can only interact with subset generalists) [61], which can lead to speciation or make species more vulnerable to extinction if their interactions are disrupted because they become dependent on a particular species for their survival.

Although changes in the physical template and biotic interactions driven by urbanization have constituted the primary focus in the study of urban evolution, the urban transition also affects eco-evolutionary changes through its profound effect on social interactions and human–natural relationships. The transition from a rural to an urban society has generated a drastic shift in the magnitude and patterns of resource use and extraction, altered the practice of farming and food production, and dramatically expanded translocations and introductions of non-native species [93]. Cities are leading the transformation of food systems and supply chains to meet rising demand by intensifying domestication and selective breeding of crops and livestock, narrowing diversity and increasing homogeneity and interdependence among countries in their food supplies. Cultivations, domestication and selective breeding associated with the global urban transition have a major impact on genetic diversity and evolutionary dynamics [94]. Furthermore, the urban transition exacerbates social disparities that both drive and are amplified by divergent eco-evolutionary outcomes within and across urban landscapes [71,95].

### (d) Space-time compression

Perhaps one of the most significant qualities that distinguishes cities from other contexts is the cultural shift and social transformation associated with increasing interactions among people [54], between people and other species [96] and among distant places [65], fostering a new wave of space–time compression [3]. Cities have served as hubs of innovation and technological advancement throughout history, owing to the expanded social interactions resulting from living in close proximity [97]. These vast technological networks that support urban regions amplify telecoupling—the interactions between distant coupled socio-economic and environmental systems—and intensify the impact of human activities on distant places [65].

Urbanization has significantly expanded the human-mediated movement of species, both intentionally and unintentionally, owing to the rapid increase in travel and commerce associated with urban areas. The success of translocated species may be affected by the urban environment through a range of mechanisms, including predator or competitor elimination, abiotic modifications or alterations to host–parasite interactions driven by changes in the composition of host communities [93,98,99]. Disrupting the relationship between host density and parasite abundance may enable introduced populations to survive at densities that would otherwise be affected by parasites [93].

The space–time compression driven by urbanization in human society is mirrored by similar phenomena occurring among wildlife and between humans and wildlife. Gilbert *et al*. [100] shows that human disturbance leads to spatio-temporal compression of species co-occurrences, which probably strengthen species interactions, with cascading effects across populations, communities and ecosystems. Cities have also dramatically increased opportunities for human–wildlife interactions, with negative consequences (e.g. conflicts, diseases, property damage and physical attacks) [101] and positive consequences (e.g. biodiversity, pest control and cultural value) [102]. Managing these interactions has important consequences for evolutionary change.

### (e) The planetary scale

The mechanisms of urban evolutionary change are most apparent at the local and regional scale, where cities and metropolitan areas transform habitat complexity and alter biotic interactions and community dynamics, all of which influence both adaptive and non-adaptive evolution. The local scale is also the scale at which most studies have been conducted and are rapidly growing. However, both the mechanisms by which urbanization affects eco-evolutionary dynamics and their impacts extend far beyond the local scale. Emissions of pollutants from anthropogenic activities associated with urbanization, such as fossil fuel use and large-scale application of pesticides, have not only altered the local ecosystem and created a distinct urban biogeochemistry in the urban landscape [60], they have also contributed to the rapid increase in the disruption of global biogeochemical cycles [103]. Urbanization is associated with high concentrations of chemicals and heavy metals and the emergence of new pollutants, such as microplastics and pharmaceuticals, that disrupt metabolic pathways and functions, driving changes in species tolerance traits. The release of synthetic chemicals in urban environments also drives antimicrobial and pesticide resistance, with global implications [104].

## 4. The eco-evolutionary dynamics of urban sustainability

Understanding how urbanization is changing evolutionary dynamics and how it will impact sustainability on a contemporary time scale demands a shift in focus from studying drivers in isolation to studying systemic properties that emerge from the underlying socio-ecological interactions. Understanding how urban eco-evolutionary dynamics affect sustainability also requires shifting our perspective from viewing urbanization as a *context* to understanding it as a *dynamic process* that alters the interactions between humans and the natural world across multiple temporal and spatial scales. Niche construction theory points out that organisms influence their own evolution and that of other organisms, both as a result of natural selection and as agents of the conditions of that selection. Therefore, organisms are both the *subjects* and the *drivers* of evolution. By transforming the environment, organisms can determine the conditions for their reproductive success. Species and their environments are intertwined in a continual feedback loop, leading to ecological and evolutionary change [105]. The emergent properties of these complex socio-eco-evolutionary systems are affected by long-term human and natural dynamics, and shape divergent ecological and human outcomes under alternative future scenarios.

### (a) Eco-evolutionary feedback and urban resilience

The evolution of species traits plays a pivotal role in shaping urban resilience [32]. As organisms adapt to urban environments, their evolving traits can alter ecosystems, thereby influencing the capacity of cities to withstand environmental change. While many species will continue to become extinct by human action, others are evolving strategies to coexist successfully within human-dominated environments. For instance, great tits (*Parus major*), a common European bird, adjust to city life through changes in genes that control their behaviour and brain development, preserving avian biodiversity and the ecosystem function they provide [106]. Similarly, urban trees, which develop resistance to air pollution and heat stress, underscore how the capacity of plants to adapt to urban stressors helps cities maintain their ecosystem function and resilience [107,108]. These trees form the backbone of urban green spaces that offer essential services, from air purification to heat mitigation and recreational spots. By altering photosynthetic, tree growth and plant defence traits, urban-driven evolutionary pressures may affect the capacity of trees to perform these functions [109]. Furthermore, evolutionary changes in many organisms involved in the carbon cycle may lead to increased atmospheric CO_2_ concentrations [110].

Importantly, the evolutionary dynamics of pests and disease vectors can pose challenges to urban resilience. The emergence of resistance in these organisms prompts the need for adaptive strategies for pest management and public health, thereby testing a city's resilience. Species evolving traits that increase tolerance to climate change-related stressors, such as heat, drought and flooding, contribute to urban resilience by supporting the functionality of GBI. For instance, the evolution of drought-tolerant grasses in urban landscapes can enhance water security in cities with water scarcity.

Evolution also governs species interactions and overall ecosystem functionality. The evolution of urban pollinators to synchronize with climate change-induced shifts in flowering times is crucial for maintaining plant biodiversity and ecosystem resilience. Evolutionary trade-offs, in which short-term beneficial traits could potentially compromise long-term ecosystem stability, emphasize the need for proactive urban management that can balance these dynamics to ensure steady provision of ecosystem services. Coastal GBI strategies often rely on the natural defence capabilities of salt marshes, which buffer wave action, slow down water and trap sediment, thereby reducing erosion and the risk of flooding. Seagrass (*Spartina* spp*.*) may adapt to urban pressures, such as pollution or altered salinity, but these adaptations could inadvertently result in morphological changes, such as reduced growth or stature, potentially compromising the wave buffering and sediment trapping capacities of the salt marsh [111].

Cities are increasingly adopting GBI and other nature-based solutions (NBS) for cost-effective environmental management, using ecosystem services to provide multifaceted benefits and foster sustainability. However, the effectiveness of these interventions can be undermined if the intricate interplay of social, ecological and technological facets of urban systems [112] and their eco-evolutionary dynamics [9] are overlooked, leading to unforeseen consequences, such as species evolving resistance, unintended evolutionary trade-offs destabilizing ecosystems or disruptions in species interactions essential to ecosystem functions that such solutions aim to preserve. Building on Sgrò *et al.* [25] I propose that harnessing our growing knowledge of urban-driven evolutionary changes and their effects on ecosystems we can foster sustainability through six primary strategies: preserving genetic diversity, promoting evolutionary potential, aiding species with evolutionary limitations, protecting evolutionary refugia, enhancing gene flow and bolstering adaptability to future environmental shifts.

Understanding the mechanisms linking eco-evolutionary changes to urban sustainability and determining when evolution may promote or inhibit organismal adaptation is crucial for predicting what trait changes are likely to occur in urbanizing environments and guiding strategies to buffer their eco-evolutionary feedback [113–115]. The limited predictability of future eco-evolutionary feedbacks underscores the need to conserve adaptive potential. Recognizing early warning indicators is crucial for averting potential adverse consequences and identifying mitigation strategies for safeguarding urban ecosystem health. The uncertainty surrounding species' adaptability highlights the importance of conserving evolutionary potential, which is the ability of a population to evolve in response to environmental change.

### (b) The eco-evolutionary dynamic of green and blue infrastructure

Green and blue infrastructure—such as parks, rivers and green roofs—are designed to mitigate the impacts of urbanization and stormwater flows, reduce heat, settle out sediment and aquatic contaminants, absorb atmospheric pollutants and improve the well-being of human residents. This infrastructure can simultaneously provide novel habitats for wildlife and reconfigure species coexistence by altering network structures and habitat composition. Understanding co-evolution in these ecological networks provides us with a key for predicting eco-evolutionary change and its potential effects on environmental and human outcomes, including biodiversity, water quality, social equity and economic cost. To adapt to and assist in mitigating climate change, green stormwater infrastructure has become a widely deployed approach in cities worldwide. Berlin was one of the earliest adopters of this approach, beginning more than 100 years ago [116]. In the US, Seattle and Boston have emerged at the forefront of developing innovative strategies for stormwater management [117], albeit with wide differences in historical deployment given the ages of the cities (Seattle was settled *ca* 1851; Boston *ca* 1630). In Seattle, the green stormwater infrastructure (built by Seattle Public Utilities and King County Wastewater Treatment Department) has managed 410 million gallons of stormwater in 2020 [118]. With innovative designs and solutions, green stormwater infrastructure presents an excellent opportunity to study evolution in hybrid human-constructed ecosystems. In particular, open-water ponds and infiltration basins play an important role in providing ecosystem functions, including regulating the microclimate, settling out sediments, providing new habitats, depositing excess runoff and pollutants, and altering the ecology, biogeochemistry and evolutionary dynamics of urban freshwater systems.

Mayors of a number of major cities across the globe, from Los Angeles to Singapore, have committed to planting a million trees in their city to mitigate climate change and increase resilience (sometimes referred to as the Million Tree Initiative). This has proven to be a remarkably effective and simple message for raising public awareness and motivating mayors to take action. Urban trees provide several environmental benefits. They absorb pollution [119], mitigate stormwater [120], cool the atmosphere [121], reduce the need for energy [122] and provide habitats for a variety of species [123]. There is also increasing evidence that trees may reduce stress and promote well-being [124,125].

**Figure 3.**
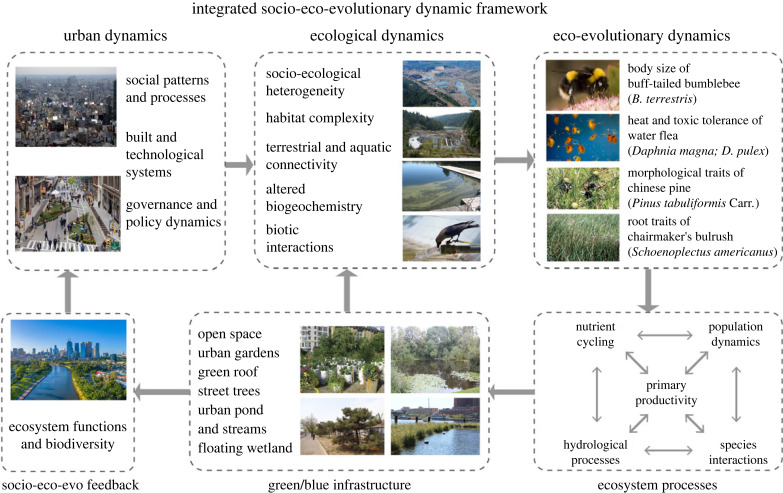
Framework linking green and blue infrastructure to eco-evolutionary dynamics. The ecological properties of urban ecosystems—including socio-ecological heterogeneity, habitat complexity, terrestrial and aquatic connectivity, modified biogeochemistry and biotic interactions—emerge from the interplay among social patterns, constructed and technological systems and governance structures. These interactions influence the dynamics of ecology and evolution, as well as ecosystem functioning, which reciprocally impact human wellbeing via the mediation of green and blue infrastructure.

However, we do not know how this infrastructure affects and is affected by the intrinsic properties of adaptive evolution of organisms. Examples of mechanisms that link GBI and eco-evolutionary dynamics are the effects of climate change, heat islands, pests and herbivory, which drive evolutionary changes in photosynthetic, tree growth and plant defence traits that affect the ability of trees to capture carbon or mitigate air pollution [82,110]. Other examples include the effects of urban heat islands on zooplankton (which might determine urban water pollution) [84], the adaptation of wetland vegetation (which may affect nutrient cycling and flood mitigation) [126] and the adaptation of marine algae and invertebrates to pollutants (which may affect marine ecosystem function and food webs) [110,127]. Because evolutionary changes in species traits may have substantial consequences on ecosystem functions, it is crucial to understand and incorporate evolutionary processes into the design and implementation of GBI (figure 3).

**Figure 4.**
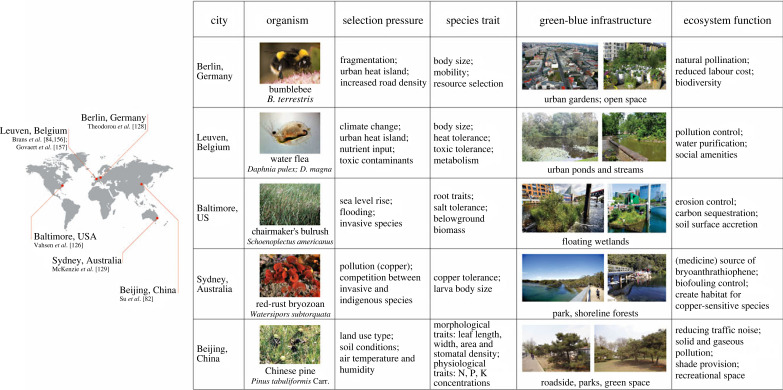
Examples of urban-driven evolutionary changes in traits that affect the ability of organisms to perform important ecosystem functions include: (i) the effect of urbanization on bumblebee body size, which affects pollination [128]; (ii) the genetic differentiation in physiology and structured pace-of-life syndromes in the water flea *Daphnia magna*, which affects water quality [84]; (iii) the effect of rapid evolution in root traits of a dominant marsh sedge on carbon accumulation and soil surface accretion, which affects marsh resilience to sea-level rise [126]; (iv) the copper-tolerance of red-rust bryozoans and its impact on local ecosystems [129]; and (v) the effects of urban land-use on leaf nutrient and morphological traits of Chinese pines, which influences their ability to mitigate atmospheric pollution and carbon emissions [82].

Figure 4 provides a few examples of urban-driven changes in organisms that affect their ability to perform important ecosystem functions. By comparing bumblebees from nine rural sites and nine cities in Germany, Theodorou *et al*. [128] shows that urbanization has an indirect positive effect on bumblebee pollination services by leading to an increase in body size. Brans *et al*. [84] analysed 13 populations of *Daphnia magna* across urbanization gradients in Flanders, Belgium, showing that urbanization drives genetic differentiation in physiology and structures the evolution of pace-of-life syndromes in the water flea. Pairing a common garden experiment of genotypes of the dominant sedge *Schoenoplectus americanus* with an ecosystem model, Vahsen *et al*. [126] show that rapid evolution in root traits of a dominant marsh sedge alters the predictions of carbon accumulation and soil surface accretion, a key determinant of marsh resilience to sea-level rise. McKenzie *et al*. [129] also show that copper-tolerant red-rust bryozoan can become a nuisance to local ecosystems. The observed adaptation of *Daphnia* to urban heat islands, toxic cyanobacteria and pesticides might determine the net effect of urban water pollution strategies [84]. Su *et al*. [82] examined trait changes of the Chinese pine (*Pinus tabuliformis* Carr.), a species that is native to China. Chinese pines along roadsides had leaves with smaller length, width and area, as well as lower stomatal density, than those growing in parks and neighbourhoods. It has been intentionally planted for the last hundred years in Beijing across an urban–rural gradient, land use types and plant developmental stages. Su *et al*. [82] found that the leaf functional traits of Chinese pine, which may affect the ability of trees to capture carbon or mitigate air pollution, have changed, although the genetic basis of these trait changes has not been determined.

## 5. Towards urban eco-evolutionary sustainability

Understanding how urbanization affects ecological resilience and stability requires expanding the concept of ecosystem complexity. In a rapidly evolving planet, *stability* is not defined as the capacity to maintain ecosystem conditions in a pre-urban or current state, or to maintain the rate of ecosystem processes despite environmental fluctuations. Instead, it is defined as the capacity of the system to adapt and evolve to include and support novel functions. In living systems, stability requires both robustness and flexibility [130]. For example, in a genetic network *robustness* is the capacity of a phenotype to overcome environmental perturbations. *Flexibility* denotes the variability in gene expression patterns throughout development and the capacity to adapt to environmental variations. From an evolutionary perspective robustness is essential for an organism to maintain the stability of phenotypic traits that are required for fitness, while flexibility allows for phenotypic innovation, enabling the organism to adapt to new environmental challenges. Torres-Sosa *et al*. [130] show that it is the interplay between conservation and innovation that drives the evolution of complex networks towards criticality, a state of dynamic systems that exhibit key properties for their evolvability. These include quick information processing, a unified response to disturbances and the capacity to assimilate a broad spectrum of external changes without altering their core functions.

In this context, robustness can be seen as: ‘the effectiveness of a system's ability to switch among multiple strategic options' [131, p. 5] that are available, to respond to perturbations. Evolution, by altering diversity, may enhance or reduce the robustness of ecological networks in urban environments. Urbanization can alter the balance between adaptive evolution and species sorting, shaping patterns of species persistence and biodiversity [9,34,35]. For instance, urbanization may impede colonization by reducing connectivity between fragmented habitats, which could allow resident species to adapt to novel conditions and monopolize resources, potentially hindering the success of new colonizers [132]. However, if urbanization enhances connectivity, species sorting may play a greater role in promoting the spatial insurance effect, whereby species track their optimal environments by shifting their ranges [133]. These outcomes may be influenced by genetic drift, particularly in small populations with limited genetic variation.

The impact of urbanization on colonization rates, genetic drift and gene flow varies across different species and cities, and can either amplify or mitigate these processes depending on the characteristics of the urban environment and the species involved. This can affect the relative importance of species sorting and evolutionary dynamics [132,133]. The ecological effects of species loss owing to urbanization on the robustness of an ecological network depend on the trophic functions of the removed species [134]. They also depend on which organisms can adapt, disperse or go extinct, and which can evolve the necessary strategies and physical characteristics to coexist with humanity [9].

Rapid evolution requires us to rethink urban biodiversity conservation and resilience from an eco-evolutionary perspective [113]. This perspective radically changes the way we think and plan cities. Understanding how city environments select for species traits will provide new insights for designers and planners to simultaneously mitigate the impact of urbanization and climate change by expanding cities' adaptive capacity while including diverse communities of people and organisms. An evolutionary perspective will help us see how historical system dynamics have shaped the system capacity for adaptation and the evolutionary potential of organisms. Evolution will affect how organisms respond to urbanization and climate change and will alter the ecosystem functions that urban sustainability depends on.

This perspective shifts the focus of planning towards human–natural interactions, adaptive feedback mechanisms and flexible institutional settings [63] to realize new cooperation between humans and the biosphere [135]. Approaching cities from an eco-evolutionary perspective allows us to broaden the dimensional space encompassing human–nature relationships, thereby unveiling potential pathways towards a new coexistence [136]. Instead of predefining ‘solutions’ that communities must implement, planning and design will rely on principles of resilience, innovation and evolvability in complex systems [73,134,137]. Evidence emerging from the study of complex systems indicates that systems with greater *heterogeneity* and *modularity* tend to have greater adaptive capacity than those characterized by highly connected, homogeneous elements [138]. Other properties of complex systems that enhance adaptive potential and foster evolutionary innovation include *cross-scale interaction*, *early warning* and *self-organization* [63,73].

Incorporating these principles into urban design and planning enhances the evolutionary resilience of populations and landscapes, thereby safeguarding critical ecosystem functions. This approach advances urban sustainability by achieving key evolutionary objectives mentioned earlier: maintaining genetic diversity, facilitating evolutionary potential, aiding species with evolutionary constraints, identifying and protecting evolutionary refugia, enhancing gene flow and strengthening adaptability to future environmental shifts (table 1; [25,27–29,136]).

**Table 1.** Principles of urban eco-evolutionary resilience^a^. (Note: principles of complexity based on Allen *et al*. [73], Scheffer *et al.* [138], Alberti [63]. Evolutionary objectives based on Sgro *et al.* [25], Smith *et al.* [27], Carrol *et al.* [28], Jørgensen *et al.* [29].)

principles	objectives						
	genetic diversity	evolutionary potential	genetically constrained mismatch	evolutionary refugia	gene flow	future socio-ecological adaptability	references
*heterogeneity*							
*heterogeneity in biological systems (from cells to ecosystems) and social systems (from communities to societies) allow system flexibility and enable them to adapt and thrive under variable conditions*	heterogeneous urban landscapes provide a variety of different microhabitats and ecological niches that support diverse species and genotypes while proving other social and economic benefits to urban populations	heterogeneous habitats support evolutionary potential by creating opportunities for different genotypes to evolve new traits to adapt to changing environments; e.g. urban gardens with a diverse array of plant species can promote the evolution of growth and defence traits better suited to new and variable environmental conditions	heterogeneity can assist the evolution of genetically constrained species (=low-evolutionary potential); e.g. in New York city the heterogeneity of urban parks provided the white-footed mouse (*Peromyscus leucopus*) a range of opportunities to adapt to the urban environment despite their low genetic diversity	unique habitats in urban environments provide species refuge from threats in the surrounding landscape. Vacant lots, wastelands, parks, and gardens may act as evolutionary refuges for unique species; e.g. vacant lots support early successional, disturbance-tolerant species that are pre-adapted to low-nutrient soils or droughts or other stressful conditions	landscape heterogeneity in urban environments provide multiple environmental gradients facilitating gene flow and selection; e.g. variation in environmental conditions within and across green spaces can facilitate gene flow among populations. Green spaces can act as stepping stones that connect otherwise isolated populations of plants or animals	urban environments can maintain genetic diversity by providing habitats for preadapting species to future conditions under climate change such as higher temperatures, altered precipitation patterns, and increased pollution; e.g. heat tolerance of some species of birds and insects and pollution tolerance of certain fungi can serve as preadaptation to climate change	[80,138–142]
*modularity*							
*modularity (selected connectivity) in populations and ecological and social networks allow information flow while maintaining autonomous functionality, and the ability to contain disturbances and avoid cascading effects*	modular populations can lead to differential selection pressures and the emergence of local adaptations which promote persistence of genetic diversity by increasing their resilience. At the community and metapopulation level, modular network interactions reduce the risk of cascading extinctions and provide opportunities for speciation and adaptation	modularity of populations and ecological networks can enhance evolvability by providing a stable framework for the evolution of new functions and interactions, and by the evolution of new traits and adaptations while maintaining existing functions and system stability	modularity of populations and genetic networks can enhance stability and robustness by limiting the diffusion of perturbations allowing evolutionary rescue	modular habitats can help maintain ‘evolutionary refugia’ by creating multiple, semi-independent compartments that can support different sets of species and ecological interactions	modular populations can facilitate gene flow between modules, which can help to maintain genetic diversity within the population. Ecological network modularity reveals critical meso-scales for biology and for conservation strategies aimed at recovering imperilled species	modularity maintains populations that may be adapted to different conditions allowing them to accumulate allelic variation that does not affect the phenotype in the present environment, but may allow adaptation in more extreme environments	[138,143–148]
*cross-scale interactions*							
*cross-scale interactions allow functional redundance across scales, add capacity under contingency and evolve innovative solutions for functional substitutions*	cross- scale interactions can increase genetic diversity within and across scales by creating opportunities for genetic exchange between populations and metapopulations	cross-scale interactions can facilitate evolutionary potential of species by providing opportunities for populations to evolve different adaptation strategies at different scales	a cross-scale approach to assisted/targeted translocation can help genetically constrained species evolve necessary strategies for survival and reproduction under changing conditions	gene flow across scales enhances benefits of evolutionary refugia by facilitating the transfer of genetic information among populations and the exchange of beneficial traits and adaptations	cross-scale interactions allow for opportunity of genetic exchange within and between metapopulations maintaining adaptive potential	cross-scale exchange of genetic information can facilitate adaptation to future conditions	[73,149–151]
*early warning*							
*early warning can anticipate catastrophic events allowing for timely response while guiding management strategies to support evolution*	monitoring genetic diversity in urban environments can help detect changes in plant and animal populations over time, such as the invasion of non-native species or declines in native populations owing to habitat loss or fragmentation allows for response before catastrophic loss	early warning and monitoring can help identify opportunities for adaptation and the evolution of new traits. For example, monitoring the genetics of a population can help detect the emergence of new alleles that may confer adaptive advantages in urban environments	monitoring can help identify and manage essential traits in low-evolutionary potential species and target assisted migrations and translocations to support evolvability	identifying and monitoring evolutionary refugia helps preserve evolutionary uniqueness	monitoring can help identify changes or disruptions to gene flow and prevent negative consequences	early warning systems can help to detect and prepare for changing environmental pressures owing to climate changes, invasive species, diseases, and other threats that negatively impact evolutionary potential	[138,151,152]
*self-organization*							
*self-organization enables natural and social systems to change their internal structure and their function in response to external circumstances*	self-organization maintains genetic variation within populations by promoting the emergence of distinct genetic groups or clusters that have unique combinations	self-organization enables the emergence of novel traits or genetic combinations that are adaptive in new environments supporting evolutionary potential	self-organization can facilitate evolutionary rescue of threatened taxa by reducing phenotypic mismatch enough that populations can sustain the costs of selection	self-organization can promote the persistence of rare and endangered species by relaxing selective pressures	speciation may be facilitated by the emergence of self-organizing barriers to gene flow (i.e. geographical isolation, behavioural differences or genetic incompatibility)	self-organization can facilitate adaptation by allowing exploration of multiple solutions to new environmental challenges leading to novel structures or behaviours that may be better adapted to future conditions	[73,153,154]

^a^Adapted form Alberti [136].

The heterogeneity of urban landscapes provides diverse microhabitats, thereby enhancing the evolutionary potential of multiple species and genotypes [139]. Designing cities with this biodiversity in mind enables different genotypes to evolve traits adaptive to fluctuating environments [140]. For instance, diverse plant species in urban gardens can catalyse the evolution of growth and defence traits better suited to urban environments [82]. Such heterogeneity also supports species with limited evolutionary adaptability, like the white-footed mouse in New York's urban parks [80]. These diverse habitats can also serve as evolutionary refugia, providing sanctuaries for unique species [141]. In cities, this may include vacant lots and gardens that can support species pre-adapted to the stresses of urban life, such as low-nutrient soils or droughts [142].

Urban design can further support eco-evolutionary feedback by implementing *a modular approach*, which acknowledges the significance of differential selection pressures and the emergence of local adaptations [143,144]. Modular landscape design enhances population stability and robustness by buffering against perturbations and providing semi-independent compartments that can sustain different species and ecological interactions [138,145,146]. This approach also allows for the conservation of ‘evolutionary refugia' and facilitation of gene flow between modules, ultimately maintaining genetic diversity within the population [147,148].

A *cross-scale design* perspective is integral to urban planning. By considering functional redundancy across scales and fostering opportunities for genetic exchange between populations, urban environments can maintain and enhance genetic diversity [73,149,150]. *Monitoring* changes in these diverse and interconnected environments is also important. Early warning systems can detect the emergence of new adaptive traits or disruptions in gene flow, and prepare for changing environmental pressures [138,151,152]. Moreover, the facilitation of *self-organization* within these environments enables natural and social systems to adapt internally to external changes, supporting the emergence of novel traits and facilitating the evolution of threatened taxa [73,153,154].

Implementing eco-evolutionary resilience principles transforms cities and their infrastructure into assets to maintain and enhance evolutionary potential, thereby enabling species to adapt to present and future rapid environmental changes.

## 6. Conclusion

In this paper I argue that integrating evolutionary principles into the planning and design of cities is critical for achieving urban sustainability. Evolutionary change plays a crucial role in ecosystem dynamics, influencing both ecosystem function and human well-being on a contemporary time-scale. By understanding the mechanisms through which urbanization affects eco-evolutionary dynamics and their feedback on ecosystem function and resilience, we can develop evidence-based strategies to promote sustainable urban development.

Knowing when and how populations can evolve will enable the protection of ecosystem function. Understanding how cities affect evolution allows us to anticipate potential ecosystem shifts. However, this task is not trivial. Predicting how urban-driven environmental change affects eco-evolutionary outcomes requires understanding how the *emergent properties* of urban landscapes alter the complex networks of ecological interactions that govern communities and ecosystem function and sustain human wellbeing.

Integrating evolutionary dynamics in urban planning and design redefines target conditions and strategies. I argue that urban sustainability must aim to maintain eco-evolutionary potential, and I propose that the cities of the Anthropocene call for expanding the time and spatial scales of urban planning and governance to include the scales of the geological and biological processes on which our planet operates.

## Data Availability

This article has no additional data.
